# Eleven quick tips for architecting biomedical informatics workflows with cloud computing

**DOI:** 10.1371/journal.pcbi.1005994

**Published:** 2018-03-29

**Authors:** Brian S. Cole, Jason H. Moore

**Affiliations:** Institute for Biomedical Informatics, Department of Biostatistics, Epidemiology, and Informatics, Perelman School of Medicine, University of Pennsylvania, Philadelphia, Pennsylvania, United States of America; Genome Quebec, CANADA

## Abstract

Cloud computing has revolutionized the development and operations of hardware and software across diverse technological arenas, yet academic biomedical research has lagged behind despite the numerous and weighty advantages that cloud computing offers. Biomedical researchers who embrace cloud computing can reap rewards in cost reduction, decreased development and maintenance workload, increased reproducibility, ease of sharing data and software, enhanced security, horizontal and vertical scalability, high availability, a thriving technology partner ecosystem, and much more. Despite these advantages that cloud-based workflows offer, the majority of scientific software developed in academia does not utilize cloud computing and must be migrated to the cloud by the user. In this article, we present 11 quick tips for architecting biomedical informatics workflows on compute clouds, distilling knowledge gained from experience developing, operating, maintaining, and distributing software and virtualized appliances on the world’s largest cloud. Researchers who follow these tips stand to benefit immediately by migrating their workflows to cloud computing and embracing the paradigm of abstraction.

## Author summary

Cloud computing has revolutionized the tech sector, but academia is slow to adopt. These 11 quick tips are geared towards helping academic researchers and their teams harness the power of cloud computing by utilizing the design patterns that have evolved in the past decade. Cloud computing can increase reproducibility, scalability, resilience, fault-tolerance, security, ease of use, cost- and time-efficiency, and much more.

This is a *PLOS Computational Biology* Education paper.

## Introduction

Cloud computing is the on-demand use of computational hardware, software, and networks provided by a third party [1]. The rise of the internet allowed companies to offer fully internet-based file storage services, including Amazon Web Services’ Simple Storage Service, which launched in 2006 [2]. Throughout the past decade, cloud computing has expanded from simple file and object storage to a comprehensive array of on-demand services ranging from bare metal servers and networks to fully managed databases and clusters of computers capable of data processing at a massive scale [3,4].

Modern cloud computing providers and the customers that utilize their services share responsibility for computer systems, with the cloud provider managing the physical hardware and virtualization software and the consumer utilizing the cloud services to architect workflows which may include applications, databases, systems and networks, storage, web servers, and much more [5,6]. In this way, cloud computing allows users to offload the burden of managing physical systems and focus on building and operating solutions.

Cloud computing has revolutionized the way businesses operate. By using a cloud provider instead of operating private data centers, companies can reduce costs by paying for only the hardware they use and only when they use it. In addition, cloud-based technological solutions offer many important advantages when compared to conventional enterprise data centers, including the ability to dynamically scale up under increased load, recover from disaster incidents automatically, remotely monitor application states, automate hardware and software deployments, and manage security through code. In addition, many cloud providers operate multiple data centers across continents, providing redundancy across different locations in the world to increase fault tolerance and reduce latency. Finally, cloud computing has evolved a new paradigm of microservice-centric application design, wherein the traditional monolithic software stack is replaced with loosely coupled components which can each be scaled individually, updated individually, and even replaced with fully managed cloud services such as message passing services, serverless function execution services, managed databases and data lakes, and even container management services. Businesses have exploited these advantages of cloud computing to gain an edge in a competitive landscape, ushering in a new era of computing that emphasizes abstraction, agility, and virtualization.

Scientific computing in academic research environments still mostly utilizes in-house enterprise compute systems such as High Performance Compute (HPC) clusters [7]. In these systems, all software, hardware, data storage, networking, and security are the responsibility of the institution, including compliance with applicable state and federal laws such as HIPAA and other regulations which govern data storage for protected health information and human genetic data. The fact that scientific institutions manage their own separate compute systems poses serious problems for reproducibility due to differences in hardware and software across institutions [8–10]. Additionally, the HPC model fails to allow researchers to capitalize on the innovations offered by cloud computing. For these reasons, we have compiled a set of eleven quick tips to help biomedical researchers and their teams architect solutions using cloud computing. We provide a high-level overview of some best practices for cloud computing with an emphasis on reproducibility, cost reduction, efficiency of development and operations, and ease of implementation.

## Templatize infrastructure with version control

Cloud computing providers such as Microsoft Azure, Google Cloud Platform, Amazon Web Services, and others have developed templating systems that allow users to describe a set of cloud infrastructure components in a declarative manner. These templates can be used to create a virtualized compute system in the cloud using a language such as JSON /or YAML, both of which are human-readable data formats [11]. Templates allow developers to manage infrastructure such as web servers, data storage, and fully configured networks and firewalls as code. These templates may be version-controlled and shared, allowing lateral transfer of full compute systems between academic institutions. Templatized infrastructure makes it is easy to reproduce the exact same system at any point in time, and this provides an important benefit to researchers who wish to implement generalizable solutions instead of simply sharing source code. Templates allow researchers to develop virtual applications that provide a control over hardware and networking that is difficult or impossible to achieve when researchers use their institutional HPC systems. Additionally, templates themselves are lightweight documents that are amenable to version control, providing additional utility. Finally, templates can be modified programmatically and without instantiating the computational stack they describe, allowing developers to modify and improve templates without invoking costs.

Version-control systems such as Git give developers immense control over software changes, including branching and forking mechanisms, which allow developers to safely implement new features and make modifications [8]. Additionally, repository hosting services such as GitHub allow researchers to share workflows and source code, aiding in reproducibility and lateral transfer of software.

In cloud computing, infrastructure of entire complex systems can be templatized. These templates can then be version-controlled, allowing researchers and developers to keep a record of prior versions of their software and providing a mechanism to roll back to an earlier version in the event of a failure such as an automated test failure. Version control therefore plays a vital role in architecting workflows on cloud computing because it applies not only to the software, but also to templates that describe virtualized hardware and networks.

Academic scientists who work in isolated compute environments such as institutional HPC clusters might not employ version control at all, instead opting to develop and operate applications and workflows entirely within the cluster. This practice is undesirable in that it fails to keep a record of code changes, fails to provide a mechanism for distribution of source code to other researchers, and fails to provide a mechanism by which collections of code can be migrated to other systems. It is strongly encouraged that absolutely every piece of code and infrastructure template be version-controlled, and further, that version control becomes a first step in all bioinformatics workflow development. Cloud computing providers often offer fully managed services for version-control hosting, allowing researchers, teams, and even whole institutions to maintain private collections of repositories without the need to manage a version-control server or use a third-party version-control service like GitHub.

An example of a cloud-based virtual appliance which uses a version-controlled template to recreate infrastructure is EVE [12]. EVE is a cloud application that utilizes snapshots of software and reference data to perform reproducible annotation of human genetic variants. The appliance’s infrastructure is declared in a CloudFormation template which can be shared, modified offline, and used to instantiate an exact copy of the same hardware–software stack for annotation, a bioinformatics workflow which is difficult to reproduce across varying compute environments that are not controlled for software and reference data versions across space and time. EVE is an example of how templatized infrastructure and imaged software and reference data allow cloud computing to enhance reproducibility of biomedical informatics workflows.

## Embrace ephemerality: Image data and software

The on-demand nature of cloud computing has driven innovation in imaging technology as well as templating technology. In contrast to local data centers, cloud computing encourages users to expand computational capacity when needed, and users do not need to leave a server running all the time. Instead, users can instantiate the hardware they need only when they need it and shut it down afterwards, thus ending the operational expense. This ephemeral approach to computing has spurred development of imaging and snapshotting services.

An important element of cloud providers is their ability to take snapshots and images of data storage volumes which can be used to later recreate the internal state of a server. A user can install software and data onto a virtual server and then create an image of the block storage devices that server uses, including the operating system, file system, partitions, user accounts, and all data. The ability to image data and software provides tremendous utility to biomedical researchers who wish to develop reproducible workflows. External data sources upon which biomedical workflows depend may change over time; for example, databases of genetic polymorphisms are updated regularly, and genome assemblies are revised as more genotype data is accrued. Imaging the reference data that is used in a particular biomedical workflow is an excellent way to provide a snapshot in time which will not mutate, providing a reproducible workflow by controlling software and data. When combined with templatized infrastructure, snapshots and images can fully recreate the state of a virtual appliance without the requirement that the end user copies data or installs and configures any software whatsoever.

## Use containers

Containers are software systems that provide the ability to wrap software and data in an isolated logical unit that can be deployed stably in a variety of computing environments [13]. Containers play an important role in the development of distributed systems by allowing tasks to be broken up into isolated units that can be scaled by increasing the number of containers running simultaneously. Additionally, containers can be leveraged for reproducible computational analysis [14]. Importantly, cloud providers often offer integration with containers such as Docker, allowing developers to manage and scale a containerized application across a cluster of servers.

A compelling example of containerized applications for biomedical informatics workflows is presented by Polanski et al., who implement 14 useful bioinformatics workflows as isolated Docker images that are provided both directly and integrated into the CyVerse Discovery Environment [15], which is an NSF-funded cyberinfrastructure initiative formerly known as iPlant [16]. These images, shared on both GitHub and DockerHub, are useful not only within the CyVerse Discovery Environment but also via managed Docker services including Amazon Web Services (AWS) Elastic Container Service, Microsoft Azure Container Service, Google Kubernetes Engine, and others.

## Manage security and privacy as code

Cloud providers often operate under a shared responsibility model for security, in which the cloud providers are responsible for the physical security of the cloud platform and the users are responsible for the security of their applications, configurations, and networks [17]. While this imposes new responsibilities on users who otherwise would operate entirely within an institutional compute system such as an HPC, it also creates opportunities to take control of security as code. Much like servers and storage volumes, firewalls and account control in cloud computing are expressed as code, which may be version-controlled and updated continuously. Cloud computing and the infrastructure-as-code paradigm allow developers to configure and deploy firewalls, logical networks, and authentication/authorization mechanisms in a declarative manner. This allows developers to focus on security in the same way as hardware and software and pushes security into a central position in the process of development and operations of cloud applications. Cloud computing also allows automated security testing, an important component of agile software development.

In addition, privacy settings are also amenable to programmatic and automated management in cloud computing. Access to specific cloud resources is controlled by provider-specific mechanisms, including role-based account management and resource-specific access control. Users are encouraged to manage privacy by a principle of minimum privilege, complying with all applicable regulations. Cloud computing providers make it easy to control which users can access which resources, including sensitive datasets. In addition, access logs for cloud-based data storage and built-in encryption mechanisms offer fine-grained auditing capabilities for researchers to demonstrate compliance.

## Use managed services instead of reinventing them

Cloud providers compete with each other to offer convenient and cost-saving managed services to perform common tasks without the user having to implement them [18]. These include message passing, email, notification services, monitoring and logging, authentication, managed databases and data lakes, cluster management tools such as for Apache Spark and Hadoop, and much more. Utilizing these services is not only cost-effective but also offloads the burden of development and maintenance. Additionally, these services are often implemented in a distributed and highly available manner, utilizing redundancy and cross-data center replication technology. All of this is provided and maintained by the cloud service provider, and effective utilization of managed services can yield tremendous gains for very little investment.

Some crucial examples of managed services which can greatly accelerate the pace of development for biomedical workflows include managed data analysis clusters such as Apache Spark. Apache Spark is a powerful and easy-to-use distributed data processing engine that has found use cases in bioinformatics, especially when working with very large datasets. Multiple major cloud providers offer a managed Apache Spark service, allowing users to skip over installing and configuring Apache Spark and even spin up an entire cluster of preconfigured Spark nodes with a few clicks. This allows scientists to go directly from raw data to distributed processing, and these services often additionally offer convenient integration with cloud storage. Another example comes in the form of managed database services, most notably Google’s BigTable and Amazon Web Service’s DynamoDB, which are both NoSQL databases that the user accesses directly through an application programming interface (API). This means that cloud users can simply put data into a database table without having to spin up a server for the database and install and manage the database itself; instead, the database is already running as a managed service in the cloud, and the user can directly call it to store and retrieve data. BigTable and DynamoDB are implemented in a distributed manner behind the scenes, providing the advantages of a high-availability system with built-in redundancy and the accompanying low latency and high durability. Using managed services like distributed computing systems and databases reduces developer burden and provides a technologically advanced solution that need not be reinvented.

An example of a cloud-based biomedical informatics workflow which benefits from managed services is Myrna, which is a pipeline for alignment of RNA-seq reads and investigation of differential transcript expression [19]. Myrna utilizes Elastic MapReduce (EMR), a managed Hadoop service offered by Amazon Web Services, as a distributed computing engine. While users could install and configure their own Hadoop environments starting from raw cloud resources, the managed service offloads the burden of configuring and managing Hadoop clusters, and has convenient features for automatic or manual scaling. Services such as EMR are great examples of ways in which cloud computing services can reduce management burden while simultaneously providing useful features that users do not need to reimplement.

## Develop serverless applications

The advent of cloud computing has spawned the creation of a new paradigm for web applications: serverless computing. Serverless computing is a model in which the user does not create and operate a web server but instead creates abstract functions that are logically connected to each other to perform all of the logic of the application. Instead of the user managing a server which runs the application’s logic, the cloud provider dynamically manages each function in real time, allocating resources and executing code. The user only pays for code that is executed and does not need to keep a web server running continuously. Additionally, serverless applications are easier to scale up because the functions which define the application’s logic are executed in isolated containers. This means that there can be many functions executing simultaneously and asynchronously without overwhelming any one server or saturating any one network environment. Serverless computing can also be blended with traditional servers instead of purely serverless applications that do not utilize any provisioned servers.

Serverless computing is a new paradigm for application development and operations that is considerably more abstract than creating and operating an application on a provisioned cloud server. In designing serverless applications, developers do not need to manage memory, application state on disk, or software dependencies. Instead, programmers define pure logic in the form of functions and the events that trigger them. This way of thinking is a challenge to adopt, but the rewards are incredible in that applications can scale without autoscaling policies that allow conventional server-bound applications to scale. Additionally, serverless applications do not continuously bill the user’s account in the way that continuous operation of a server would. Finally, serverless computing may reduce development time and cost by removing the responsibility of managing servers and their resources from the developer.

As an example, Villamizar et al. recently implemented a real-world web application in a traditional monolithic design, a user-managed microservice design, and a fully serverless design which uses AWS Lambda functions [20]. Cost comparisons showed that the serverless implementation reduced costs by over 50% while simultaneously providing agility and fine-grained scalability within the application. While serverless computing is a new paradigm that has yet to see widespread adoption in biomedical informatics, this example illustrates the capability of serverless applications to transform the way biomedical informatics workflows are developed and operated.

## Be agile: Iterate with small releases

Agile development is an emerging set of principles for software development and deployment that emphasizes flexibility, small releases, and adaptivity to change. Instead of focusing on large releases with monolithic changes to large features in a software application, agile teams focus on a nearly continuous stream of small updates. This allows teams to respond to changes in project scope, design criteria, and process changes more effectively than while building toward a major release. Additionally, agile development has brought special emphasis to techniques such as automated testing, continuous integration and delivery, and test-driven development.

Cloud computing is a great fit for agile development, and agile development is a great fit for cloud computing. With cloud computing, deploying new servers and calling new managed services is fast, allowing developers and teams to iterate quickly. Cloud computing offers developers fast and on-demand access to a variety of different testing environments, which can aid in automated testing and blue-green deployments for uninterrupted services during updates. In addition, many cloud providers offer managed services for continuous integration and continuous delivery. These services can automatically build, test, and deploy software every time a change to the source code is made. In many ways, the agile paradigm can enhance productivity for biomedical research teams, and cloud computing offers many avenues for agile development.

## Embrace abstraction: Decouple components

Decoupling is the process by which separate components of a system are rendered less interdependent. For example, an application which utilizes a database and a web server can benefit from migrating the database to a different virtualized server. The decoupled database and web server can then be individually updated and maintained without affecting each other. In addition, the database and web server components can be individually scaled and extended, imparting elasticity into the entire application. Finally, the decoupled system is less fault-tolerant and less prone to resource competition, including processors, memory, disk read/write, and network throughput.

Decoupled systems are modular in their nature, and cloud computing provides the ability to decouple components through message passing, virtualized networking capabilities, and managed services. For example, database tiers can be replaced by managed database services. This allows total decoupling of the database and the web server in the above example, so if one experiences a fault, the other is not affected. Additionally, a conventional database tier can be maintained but on a separate server or group of servers than the web server itself, and virtualized networking can allow these two components to access each other over the same subnet. Some cloud providers even offer managed services to design and operate decoupled systems, allowing developers to focus on the components without having to design message passing and handling logic. Decoupled systems are an important part of design best practice for highly available cloud architectures and as such are an active area of development.

## Utilize built-in sharing mechanisms provided by cloud providers

Cloud providers often offer mechanisms by which researchers can share components of cloud systems simply by making them public instead of private. For example, images of servers and snapshots of storage volumes can be made public by changing their permissions. Additionally, single users can be added without making the image or snapshot public, for example to provide the ability for a peer reviewer to access components of a cloud system without opening it to the general public. In another example, datasets stored in cloud-based object storage can be shared with specific user accounts or made generally public. Examples of this include the Cancer Genome Atlas and the 1000 Genomes Project, both of which offer publicly available data which utilizes cloud storage.

Researchers and developers can also develop templates of cloud systems which utilize snapshots and images that are then made public, allowing other users to instantiate perfect copies of a reproducible computing environment. An example is a researcher who architects a workflow, then saves a snapshot of the storage volume that contains installed and configured software alongside any reference datasets used. The researcher can then create a template that references these images and make that public, thereby creating a fully reproducible virtual application that has tremendous advantages over simply disseminating source code and referring to versions of publicly available datasets. The ability for components of cloud systems to be shared simply by changing settings to allow specific or general access is an advantage of cloud computing.

## Proactively manage costs and budgets

In contrast to traditional academic compute systems which are constructed under large, up-front capital expenses, cloud computing requires little or no up-front cost and is billed as a recurring operational expense. Users of cloud computing services are billed for what they use, often on a monthly cycle. This shift in billing methods can lead to researchers being shocked with a bill that is much higher than anticipated. It is the responsibility of the user to track expenses in real time, manage costs, and adhere to budgets, which is often not a concern that academic compute users have had to monitor, especially when academic compute systems are entirely covered by indirect costs. In addition, cloud computing providers often charge for services that academics are not used to paying for, such as data transfer and storage. These unexpected costs, coupled with the operational expense nature of cloud computing, can result in researchers receiving “bill shock” at the end of the first period of active cloud computing utilization.

Cloud providers have developed several mechanisms by which users can manage costs and budgets. First, most major cloud providers have services that provide a real-time breakdown of expenses categorized by service type, including data transfer, data storage, networking expenses, compute time, managed services, and more. In addition, cloud providers offer budget calculators such as the AWS Simple Calculator that allow users to estimate costs before launching any services. Finally, some cloud providers offer full budget management suites such as AWS Budgets which allows users to set custom alerts for budget thresholds and provides usage-to-date monitoring functionalities to maintain a tight command over spending. While the use of cloud services to monitor budgeting and expenses requires extra effort on behalf of the user, new features such as daily and per-minute quotas offered by Google Cloud Platform’s App Engine offer fine-grained control over cost management by setting hard limits on resource utilization.

While academic computing is often covered under indirect costs of grant funding, cloud computing invokes expenses as it is used, providing the opportunity for users to lose track of their spending rate. However, diligent and regular utilization of built-in budget and cost-management tools is a necessary part of cloud computing. In addition to cost-management and budget tools, government research sponsors such as NIH and NSF have launched cloud computing initiatives such as CyVerse to speed adoption of cloud computing in academia [16]. Finally, cloud providers themselves often provide free credits for researchers, such as the AWS Cloud Credits for Research award and the Microsoft Azure for Research program.

## Dive into new cultures

Much of the activity in the cloud computing ecosystem takes place outside of the realm of academic research. The tech community hosts a diverse series of conferences ranging from massive international gatherings such as re:Invent to distributed, local meetups such as Python User Groups, data science groups, and DevOps Days. The latter is an example of a conference in which scientists have the opportunity to present their research and development and simultaneously interact with leading technologists, from whom scientists and researchers can benefit by exposure to the latest tools and design patterns that are driving innovation in the tech sector but have yet to reach adoption in academia. In addition to conferences and meetups, much of the discussion of technological advances in cloud computing takes place on social media platforms. In both cases, scientists stand to benefit by interfacing and interacting with the tech sector and may find a lot more common ground than expected. Developers and engineers in the tech sector are often keenly interested in scientific research, and if scientists and academics can immerse themselves into the tech culture instead of merely attending scientific conferences and meetings, substantial mutual benefit may be obtained. Finally, tech meetups and conferences are a great way to network and source new talent with accompanying new ideas and cutting-edge skills.

## Limitations and future directions

Cloud computing offers the potential to completely transform biomedical computing by fundamentally shifting computing from local hardware and software to on-demand use of virtualized infrastructure in an environment which is accessible to all other researchers. However, many challenges and barriers to adoption remain pertinent to biomedical informatics and other scientific disciplines. Existing software and code bases might not easily migrate from academic computing centers to cloud providers, and performance of existing software might be negatively impacted by deployment in the cloud; for example, network latency between filesystem and CPU and network bandwidth between database and application tiers could be considerably slower in a cloud deployment when compared to a single data center. To mitigate this and ease transition from local to cloud deployment, containerization systems such as Docker and Kubernetes are promising candidates for software deployment in diverse environments. In addition to migration, cloud computing utilizes a different billing model when compared to academic computing centers, which are often funded by large, up-front capital expense with recurring expenses that might be covered by indirect costs. In contrast, cloud computing providers frequently require no up-front capital expense, and instead, users are billed for on-demand uses purely as operational expenses. This can result in surprise “billing shock” when users are taken off-guard with a monthly bill that is much higher than expected. While use of cloud computing services for cost management is an advisable use pattern, it’s the user’s responsibility to proactively manage costs and maintain a budget in real time. Finally, while academic computing centers are often compliant with government regulations concerning sensitive data such as protected health information, cloud computing can present a considerable privacy and security risk when used in a manner which compromises data privacy, for example by accidentally making data publicly accessible by changing privacy settings via a cloud provider’s web console. In academic computing, users have no control over the firewall and authentication/authorization of the compute system, but in the cloud the user is entirely responsible for data privacy and security for systems they create and utilize. This shift in responsibility is a grave concern for users with sensitive, protected, and regulated data, and users of cloud computing must manage their own compliance with international, federal, state, and local laws. An example of this is the Database of Genotypes and Phenotypes, which recently has added cloud computing to the Authorized Access mechanism for research use of deidentified genotype and phenotype data [21].

As cloud computing technology continues to innovate at a rapid pace, the future holds exciting possibilities for biomedical informaticians. The pace of data acquisition in biology and medicine continues to increase at an unprecedented rate, and the vertical and horizontal scaling capabilities of cloud computing are an ideal fit. Despite this, the concerns for privacy and security in biology and medicine demand the advent of managed services specifically tailored to clinicians and researchers. While cloud providers are responsible for security of the physical hardware and the underlying software used to provide cloud computing services to the end user, users are responsible for data security and privacy for infrastructure they provision and use. For this reason, advances in cloud security and privacy for sensitive data are needed to bridge the gap between on-premise academic compute environments, which often have their own dedicated IT staff, and cloud environments, where no such staff currently exists at many institutions and universities. In addition to security and privacy concerns for the future of biomedical cloud computing, education and academic support for cloud computing is an area which can benefit from increased investment and development on behalf of cloud providers, academic institutions, grant-funding agencies, and individual research groups.

## Conclusion

Cloud computing holds the potential to completely change the way biomedical informatics workflows are developed, tested, secured, operated, and disseminated. By following these 11 quick tips, researchers can ease their transition to the cloud and reap the rewards that cloud computing offers.

## Supporting information

S1 Text Glossary of terms. Terminology used in the manuscript is defined.
(DOCX)
