# Supplementary material for: Eleven quick tips for architecting biomedical informatics workflows with cloud computing
Source: PLoS Comput Biol. 2018 Mar 29;14(3):e1005994. doi: 10.1371/journal.pcbi.1005994 (PMC5875741; doi:10.1371/journal.pcbi.1005994)
Supplement: S1 Text — Terminology used in the manuscript is defined. (DOCX) [file pcbi.1005994.s001.docx]

**Glossary**

Cloud computing: The on-demand use of computational hardware, software, and networks provided by a third party

Simple Storage Service: The first cloud computing service offered, often abbreviated S3. This object storage system was launched in 2006 by Amazon Web Services and continues to function today.

Public cloud: A cloud computing provider which owns and operates the underlying hardware, virtualization software, and data centers. Contrast to private enterprise solutions such as academic high performance compute clusters and corporate data centers which are owned, maintained, and operated by the academic institution or corporation.

Microservice: A component of an application which is loosely coupled to other applications components such that the application becomes resilient to failure and capable of scaling at the individual component level.

High Performance Compute cluster: Often abbreviated HPC, a high performance compute cluster is a networked array of individual computers used to execute computational operations in parallel. Often managed by a scheduler or grid system which dispatches jobs to worker nodes connected by networking infrastructure.

Infrastructure: Hardware used in computing, including physical computers, networking devices, storage and storage arrays, and virtual components such as virtualized firewalls and virtual machines.

JSON: JavaScript Object Notation is an open standard, language-independent, descriptive format for describing objects. Among a variety of uses, JSON is used in internet communication, data serialization, software configuration, and as a template language for cloud computing resources, including Microsoft Azure Resource Manager.

YAML: YAML Ain’t Markup Language is an alternative to JSON based on the Perl programming language’s data types. In contrast to JSON, YAML allows whitespace indentation instead of braces and brackets to denote structure. Both YAML and JSON can be used for templating by the CloudFormation templating system offered by Amazon Web Services, and YAML is supported by Google Cloud Deployment Manager.

Template: A text document that declares resources to be provisioned by a cloud service provider.

Containers: Software encapsulation mechanism in which the operating system, an application, and the application’s dependencies are wrapped in a container which is run by a server in an isolated environment. Containers are virtual environments at the operating system level, making them more lightweight than virtual machines. Container systems such as Docker have become popular for development and operation of distributed systems.

Managed Service: A functionality or software component offered by a cloud provider which customers can utilize instead of implementing themselves. Managed services vary between cloud providers but can ease burden of development and maintenance by providing an out-of-box solution for common design patterns such as message passing, monitoring and logging resources, deploying additional servers to an application layer or database layer (autoscaling), authentication and authorization services, network routing and Domain Name Services, and more. Managed Services are often implemented in a highly available or distributed manner.

NoSQL: Any non-relational database. By abandoning the tabular schema and Structured Query Language (SQL) of relational databases, NoSQL databases allow flexible data storage and retrieval. Many NoSQL databases are popular for use in distributed systems and also offered as managed services.

Serverless computing: A computing model in which a cloud computing provider manages execution and resource allocation of a program. Servers are still used in serverless computing, but are not managed by the application developer, and instead are managed entirely by the cloud provider, allowing the developer to forego resource management entirely.

Agile software development: A software development methodology that emphasizes quick and flexible adaptation to change through small releases, among other values and principles.

Decoupled Components: Components of a software are decoupled when they are not interdependent and change in one component does not require change in another.
